# Human Mitochondrial Ribosomal RNA Modification-Based Classification Contributes to Discriminate the Prognosis and Immunotherapy Response of Glioma Patients

**DOI:** 10.3389/fimmu.2021.722479

**Published:** 2021-09-09

**Authors:** Peng Wang, Jingying Li, Miaojing Wu, Minghua Ye, Kai Huang, Xingen Zhu

**Affiliations:** ^1^Department of Neurosurgery, The Second Affiliated Hospital of Nanchang University, Nanchang, China; ^2^Institute of Neuroscience, Nanchang University, Nanchang, China; ^3^Comprehensive Intensive Care Unit, Second Affiliated Hospital of Nanchang University, Nanchang, China; ^4^East China Institute of Digital Medical Engineering, Shangrao, China

**Keywords:** mt-rRNA, tumor microenvironment, genomic variation, immunotherapy, temozolomide, glioma

## Abstract

**Background:**

Epigenetic regulations of the tumor microenvironment (TME) and immunotherapy have been investigated in recent years. Nevertheless, the potential value of mitochondrial ribosomal RNA (mt-rRNA) modification in regulation of the TME and immunotherapy remains unknown.

**Methods:**

We comprehensively investigated the mt-rRNA-modification patterns in glioma patients based on nine regulators of mt-rRNA. Subsequently, these modification patterns were correlated systematically with immunologic characteristics and immunotherapy. An “mt-rRNA predictor” was constructed and validated in multiple publicly available cohorts to provide guidance for prognosis prediction and immunotherapy of glioma patients.

**Results:**

Two distinct patterns of mt-rRNA modification were determined based on the evidence that nine regulators of mt-rRNA correlated significantly with most clinicopathologic characteristics, immunomodulators, TME, immune-checkpoint blockers (ICBs), and prognosis. Patients with mt-rRNA subtype II presented significantly poorer overall survival/progression-free survival (OS/PFS), but higher tumor mutational burden (TMB), more somatic mutations, and copy number variation (CNV). These two mt-rRNA subtypes had distinct TME patterns and responses to ICB therapy. An mt-rRNA predictor was constructed and validated in four glioma cohorts. The subtype with high mt-rRNA score, characterized by increased TMB, infiltration of immune cells, and activation of immunity, suggested an immune-activated phenotype, and was also linked to greater sensitivity to immunotherapy using anti-programmed cell death protein 1 (PD-1) but resistance to temozolomide.

**Conclusions:**

Regulators of mt-rRNA modification have indispensable roles in the complexity and diversity of the TME and prognosis. This novel classification based on patterns of mt-rRNA modification could provide an effective prognostic predictor and guide more appropriate immunotherapy/chemotherapy strategies for glioma patients.

## Introduction

Gliomas are the most common primary intracranial tumors of the central nervous system. Of the subtypes of gliomas, glioblastomas are the most malignant and deadliest (1–3). The previous treatment options for gliomas (maximal resection, adjuvant chemotherapy with temozolomide, and radiotherapy) have failed to achieve satisfactory results (4). Developments in epigenetics and immunology have enabled molecular-targeted therapies and immunotherapies for gliomas. However, most of these potential new therapies are being tested in clinical trials and have not been found to significantly lengthen the survival of patients suffering from glioma (5, 6). Therefore, the exploration of novel therapeutic strategies on glioma is a long-term and arduous task.

More than 170 types of RNA modifications have been reported. These modifications are present in all living organisms and have indispensable roles in biological activities, with post-transcriptional modifications of ribosomal RNA accounting for a large proportion (7). Mammalian mitochondria possess their ribosomes. The latter consist of two subunits (large and small), which synthesize the 13 key proteins of the oxidative-phosphorylation system (8–10). Hence, homeostasis of the biogenesis and modification of mitochondrial ribosomes are essential for cellular metabolism and mitochondrial translation. Abnormal mitochondrial ribosome modification can lead to the interruption of mitochondrial protein synthesis and impedes assembly of the components of the mitochondrial respiratory chain, which usually leads to metabolic-related diseases (11, 12). With the development of cryo-electron microscopy, nine modifications of mt-rRNA have been identified. All corresponding modifying enzymes have been described to explore their effects on the biogenesis and function of mitochondria (9, 10, 12, 13). The prominent modification of mammalian mt-rRNA involves catalysis by methyltransferases consisting of TRMT2B (modifies the nucleotide m^5^U429), METTL15 (modifies the nucleotide m^4^C839), NSUN4 (modifies the nucleotide m^5^C841), TFB1M (modifies the nucleotide ${\text{m}}_{2}^{6}$A936/7), TRMT61B (modifies the nucleotide m^1^A947), MRM1 (modifies the nucleotide Gm1145), MRM2/FTSJ2 (modifies the nucleotide Um1369), MRM3/RNMTL1 (modifies the nucleotide Gm1370) and RPUSD4 (modifies the nucleotide Ψ1397) (11, 12, 14–16). Deeper understanding of these regulators could aid the determination of the function and mechanism of mt-rRNA modification in post-transcriptional regulation. Accumulating evidence indicates that expression disorders and genetic variations of mt-rRNA regulators are associated with developmental defects, apoptosis, cardiomyopathy, metabolic disorders, progression of malignant tumors, and immunomodulatory abnormalities (17–19).

Immunotherapy is represented by immune-checkpoint blockade. It has been studied extensively in recent years as novel and satisfactory therapy for malignant tumors. Unfortunately, immunotherapy cannot extend the survival of patients (20, 21). Numerous studies have revealed that the microenvironment of tumor cells, rather than the genetic and epigenetic variations of tumor cells, plays an essential part in the occurrence and malignant progression of tumors (22). Further study of the tumor microenvironment (TME) has revealed its important role in tumor progression, immune escape, and immunotherapy response. Anti-programmed cell death protein 1 (PD-1) and its ligand (PD-L1) are the most studied and most efficacious immune-checkpoint blockers (ICBs). They mainly regulate the immune response of T cells in the TME so as to avoid anti-tumor immune reactions, and then attack the tumor (21, 23). Anti-PD-1 has been used in the treatment of melanoma, non-small-cell lung cancer, and colon cancer, but its clinical efficacy against glioma is not high. Several studies have revealed that the relatively high immunosuppressive microenvironment and low tumor mutational burden (TMB) may lead to immunotherapy failure (24–26). Therefore, the selection of suitable immunotherapy for glioma patients through comprehensive analyses of transcriptional regulations, genetic variations, and the immune microenvironment of gliomas may be challenging.

In this study, we analyzed the expression patterns and immunological value of the regulators of mt-rRNA modification. Based on the features of nine mt-rRNA regulators, we identified two patterns of mt-rRNA modification in glioma patients which had distinct functional annotations, clinicopathologic characteristics, and survival outcomes. Subsequently, integrated analyses revealed significant differences in genomic variation, the TME, and ICB expression levels between the two subtypes of patients, and determined the different responses to immunotherapy. Therefore, we constructed an “mt-rRNA predictor” that could distinguish the two mt-rRNA subtypes of glioma patients and verified it in four independent cohorts of glioma patients. This mt-rRNA predictor could provide a selection strategy for screening glioma patients who elicit a positive response to immunotherapy or temozolomide therapy. In this way, we aimed to provide novel ideas for survival prediction and better targeted therapy for glioma patients according to the classification of mt-rRNA modification patterns.

## Materials and Methods

**Figure 1:**
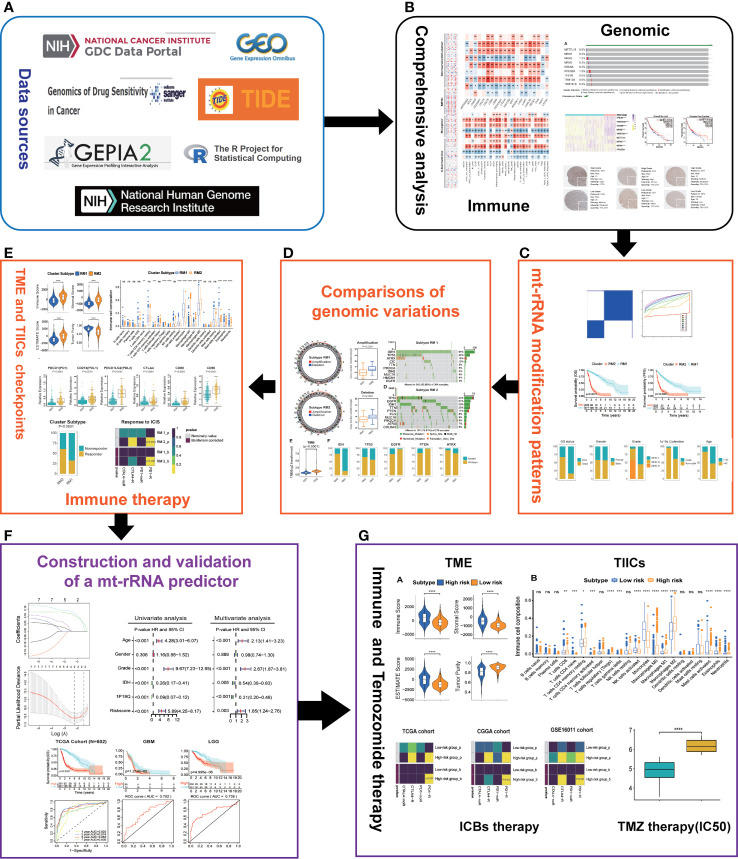
Overview of this study. **(A)** Data sources and analytic software used in this study. **(B)** Comprehensive analyses of nine regulators of mt-rRNA modification. **(C)** Identification of the patterns of mt-rRNA modification. **(D)** Comparisons of genomic variations between two patterns of mt-rRNA modification. **(E)** Correlations between the patterns of mt-rRNA modification and immunologic elements and immunotherapy. **(F)** Construction and validation of a mt-rRNA predictor and the prognostic role of this mt-rRNA predictor in glioma. **(G)** Role of this mt-rRNA predictor in predicting the response to ICB immunotherapy and temozolomide chemotherapy. Significance: ns, *P* > 0.05, *P < 0.05, **P < 0.01, ***P < 0.001, ****P < 0.0001.

The flowchart of this work is shown as **Figure 1**.

### Acquisition and Preprocessing of Datasets From Glioma Patients

Data on RNA-sequencing, somatic mutations, and copy number, as well as the matched clinical data of glioma patients, were obtained from The Cancer Genome Atlas (TCGA; https://portal.gdc.cancer.gov/), Chinese Glioma Genome Atlas (CGGA; www.cgga.org.cn/) and Gene Expression Omnibus (GEO; www.ncbi.nlm.nih.gov/geo/) databases. After excluding patients with incomplete clinical information, 1136 glioma samples (TCGA, n = 602; CGGA, n = 286; microarray data (GSE16011), n = 248) were gathered for further analyses. Subsequently, transcripts per kilobase million (TPM) values of RNA-sequencing data, robust multichip averaging analysis (RMA)-processed values of microarray data (GSE16011), RNA-sequencing data (RSEM value), and real-time reverse transcription-quantitative polymerase chain reaction (RT-qPCR) data (SAHNU cohort) of clinical samples were log2-transformed and normalized to make the gene-expression profiles of different platforms comparable. The clinical information of the 1136 public glioma patients is displayed in **Supplementary Table S1**. Furthermore, the somatic-mutation data and frequencies of genes (in mutation annotation format) were analyzed using “maftools” within R (R Institute for Statistical Computing, Vienna, Austria; www.r-project.org/). Amplification or deletion of copy number variation (CNV) data were identified using the GISTIC algorithm (27). Moreover, the half-maximal inhibitory concentration (IC50) of Temozolomide in glioma patients and matched RNA-sequencing data were downloaded from the Genomics of Drug Sensitivity in Cancer (GDSC) database (www.cancerrxgene.org/) (28).

Frozen specimens of glioma used in the present study were obtained from patients who underwent surgery at The Second Affiliated Hospital of Nanchang University (SAHNU) from 2015 to 2020. Clinical information was obtained through electronic medical records. Overall survival (OS) data were not available because information based on telephone follow-up were missing. Thirteen samples of high-grade glioma (World Health Organization (WHO) grade III, IV), 12 samples of low-grade glioma (WHO grade II), and eight samples of non-neoplastic brain tissue (NBT; collected from surgery of people with intractable epilepsy, and which were used as controls) were employed. The clinical information of these 33 patients is also displayed in **Supplementary Table S1**.

### Ethical Approval of the Study Protocol

The study protocol was approved by the medical ethics committee of the Second Affiliated Hospital of Nanchang University (Nanchang, China) and was undertaken in accordance with the Helsinki Declaration 1964 and its later amendments. Written informed consent was acquired from each glioma patient.

### Immunohistochemistry

The immunohistochemistry of pathologic specimens of the nine regulators of mt-rRNA modification were downloaded from The Human Protein Atlas (www.proteinatlas.org/). Meanwhile, the quantity and intensity of Staining as well as the information of patients were acquired online.

### Immunological Features of the TME in Glioma

The immunological features of the TME in glioma were evaluated: expression of immunomodulators; abundances of immune cells and stromal cells; infiltration level of tumor-infiltrating immune cells (TIICs); ICBs expression level. First, we obtained the composition of 122 immunomodulators (immune stimulators, major histocompatibility complex molecules, chemokines, and receptors) from the research of Charoentong et al., and collected 28 ICBs with therapeutic potential from the work of Auslander and colleagues (29, 30). The ESTIMATE algorithm was utilized to evaluate the abundance of immune cells and stromal cells, as well as the tumor purity based on the expression profiles of glioma patients (31). Four types of scores were generated by the ESTIMATE algorithm: positively reflecting the abundance of stromal cells (stromal score), positively reflecting the abundance of immune cells (immune score), positively reflecting nontumor composites (ESTIMATE score), and tumor purity. We also calculated the infiltration level of 22 types of TIICs in the TME based on the expression profiles of glioma samples using a deconvolution method according to linear support vector regression (CIBERSORT) (32). Thereafter, the enrichment score of 29 “immune signatures” (including the types, functions, and molecular pathways of TIICs and the immune activity of tumors) were quantified using single-sample gene-set enrichment analysis (ssGSEA) according to the method described by Xu and colleagues (33).

### Unsupervised Clustering of mt-rRNA Regulator-Based Classification of Glioma Patients

The expression levels of nine regulators of mt-rRNA modification were extracted from the TCGA dataset for further classifying (34). Unsupervised clustering analysis based on a machine-learning algorithm (k-means) was undertaken to identify distinct patterns of mt-rRNA modification according to the expression profiles of nine mt-rRNA regulators, and we classified glioma patients for further evaluation. The number of clusters and their stability was determined comprehensively by the steps mentioned above as well as 1000-times repetitions, the relative change in the area under the cumulative distribution function (CDF) curve, and the consensus “heatmap” based on the “ConsensusClusterPlus” package in R (35). We further explored the correlation between clinicopathologic features and mt-rRNA subtypes of glioma patients. Survival analyses using the Kaplan–Meier method were used to investigate the prognosis of glioma patients with different mt-rRNA subtypes: OS and PFS.

### TMB and CNV Between Two mt-rRNA Subtypes

Maftools and GenVisR were run to analyze and visualize the mutation types and frequencies of genes between two mt-rRNA subtypes (36, 37). The TMB is the total number of nonsynonymous mutations and is an emerging biomarker for the response to immunotherapy. The TMB was compared among different subtypes of mt-rRNA-modification patterns. In addition, significant amplifications and deletions in the whole genome were identified and visualized further using “RCircos” within R (38).

### Functional Annotation and Gene Set Variation Analysis

We wished to explore the significantly enriched molecular pathways of the different subtypes of mt-rRNA-modification patterns. Hence, we undertook GSVA (a non-parametric and unsupervised method) using the R package “GSVA “based on the gene sets of “c2.cp.kegg.v6.2.symbols”, which we downloaded from the Molecular Signatures Database (MSigDB) database (www.gsea-msigdb.org/gsea/msigdb/) (39). The KEGG pathways with an adjusted P < 0.05, |log2 fold change (FC)| > 0.1 and false discovery rate (FDR) < 0.05 were considered significant between the two mt-rRNA subtypes.

### Prediction of ICB Therapy Response

Tumor Immune Dysfunction and Exclusion (TIDE; http://tide.dfci.harvard.edu/) is an algorithm used to determine the characteristics of T-cell dysfunction by testing how the expression of each gene in a tumor interacts with the level of cytotoxic T lymphocyte (CTL) invasion to affect the survival of patients and response to immunotherapy (40). We predicted the clinical response of glioma patients to immunotherapy using the TIDE algorithm based on the expression profiles in each cohort. Subsequently, the clinical response to ICB therapy was also predicted with the subclass mapping (https://cloud.genepattern.org/gp/) method (41). Bonferroni-corrected P < 0.05 was considered to be a significant response or non-response to therapy using anti-PD1 or anti-CTLA4 with the cutoff FDR < 0.05.

### Construction and Validation of the mt-rRNA Predictor

The total of 1136 glioma patients were classified into a training set (TCGA) and three validation sets (CGGA, GSE16011, SAHNU). First, in the training set, we undertook univariate Cox analysis of these mt-rRNA regulators using the R package “survival”. Subsequently, the least absolute shrinkage and selector operation (LASSO) algorithm was utilized to select optimal-candidate differentially expressed mt-rRNA regulators with the best discriminative capability in the training set. Then, we constructed an mt-rRNA predictor based on the expression profiles of mt-rRNA regulators weighted using the multivariate Cox regression coefficient as follows:

$$mt-rRNA\;score=\sum_{i=1}^{n}\left(Coef_{i}+Exp_{i}\right)$$

where *Coef_i_* is the coefficient and *Exp_i_* is the expression of mt-rRNA-modification regulators. The coefficient of each regulator was also obtained to calculate the mt-rRNA score in the validation sets. Specifically, glioma patients were classified into low-score and high-score subtypes based on the median mt-rRNA score. The prognostic importance of the mt-rRNA predictor was assessed between the two subtypes using the Kaplan–Meier method, and the prediction efficiency was tested further with receiver operating characteristic (ROC) curves. Moreover, univariate and multivariate Cox regression analyses were carried out to explore the prognostic value of the mt-rRNA predictor with multiple clinical and molecular pathologic characteristics. Similar methods were used to verify the predictive performance of mt-rRNA predictor in the validation sets.

### Chemotherapeutic Response to TMZ

Temozolomide is first-line chemotherapy for glioma patients. We obtained the IC50 of temozolomide in glioma specimens and RNA-sequencing data from the largest publicly available pharmacogenomics database (GDSC) and normalized them by log2-transformation for further analyses. Glioma specimens treated with temozolomide were divided into two groups according to the median mt-rRNA score.

### Quantitative Real-Time Polymerase Chain Reaction of the SAHNU Cohort

We used methods we described previously to measure mRNA expression of regulators of mt-rRNA modification in glioma specimens (42). Briefly, total RNA was extracted from brain tissue and reverse-transcribed into complimentary-DNA. Next, relative mRNA expression of genes was normalized to that of GAPDH, and we evaluated the fold change using the 2−^ΔΔCT^ method. The primer sequences used for RT-qPCR were obtained from RiboBio (Guangzhou, China) and were as follows: MRM2 forward 5’-GTGATTCTGAGCGACATGGC-3’, reverse 5’-ATGACTCTTTCCTGCTGGCT-3’; TRMT2B forward 5′-TCAAGAGTCCTAAATGCACAACC-3′, reverse 5′-CCAGGAGTCATCTCTACAATGC −3′.

### Statistical Analyses

Correlations between variables were assessed using Spearman or Pearson correlation analyses. Variables with a normal distribution were analyzed by the unpaired Student’s t-test. Variables with a non-normal distribution were analyzed by the Mann–Whitney U-test. For comparisons of categorical variables, the Kruskal–Wallis test and one-way ANOVA were used for non-parametric and parametric methods, respectively. The Kaplan–Meier method was employed to calculate the survival curve of categorical variables, whereas the log-rank test was used to estimate significance. According to the method of Hoshida and colleagues, the Bonferroni correction was applied to correct nominal P-values in the subgroup analysis of ICB immunotherapy. Statistical analyses were carried out using R 3.6.5, and P < 0.05 (two-sided) was considered significant.

## Results

### The Value of Nine mt-rRNA Modification Regulators in Gliomas

**Figure 2:**
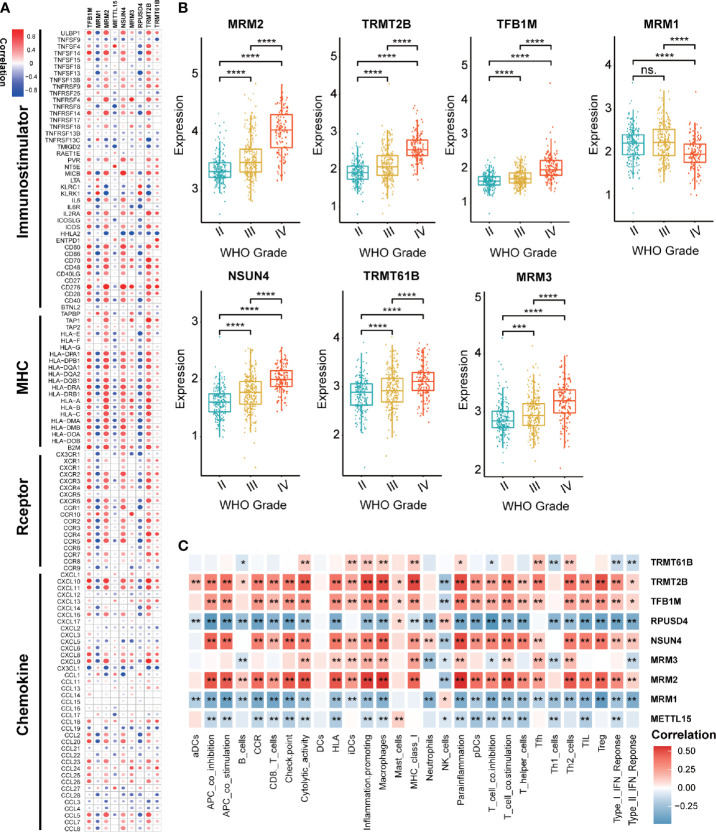
Value of regulators of mt-rRNA modification in gliomas. **(A)** Correlation between nine regulators of mt-rRNA modification and 122 immunomodulators. **(B)** Expression of each dysregulated mt-rRNA-modification regulator. **(C)** Correlation between nine mt-rRNA-modification regulators and 29 immune signatures. Significance: ns P > 0.05, *P < 0.05, **P < 0.01, ***P < 0.001, ****P < 0.0001.

Considering the possible biological functions of mt-rRNA-modification enzymes in gliomas, we conducted a comprehensive study on these regulators in TCGA cohort. As shown in a heatmap, the expression of most regulators of mt-rRNA modification was associated significantly with the WHO grade (**Supplementary Figure S1A**). Measurement of expression of each dysregulated mt-rRNA-modification regulator showed MRM1, TRMT2B, TFB1M, MRM3, NSUN4, TRMT61B, and MRM3 to be correlated significantly with each WHO grade (**Figure 2B**). Moreover, we observed protein expression of MRM1, MRM3, TRMT2B, and NSUN4 to be significantly different between low- and high-grade gliomas, whereas other regulators could not be obtained due to lack of data (**Supplementary Figures S1B–D**). Afterwards, we undertook survival analyses (OS and PFS) using the GEPIA2 website. Glioma patients were stratified into low- and high-expression groups based on the median expression of each regulator of mt-rRNA modification, respectively. Survival analyses using the Kaplan–Meier method indicated that these regulators of mt-rRNA modification were prognostic biomarkers in OS and PFS (P < 0.01 for all) (**Supplementary Figure S2A**), except for METTL15 (**Supplementary Figure S2B**). Furthermore, we observed that the genomic alterations (somatic mutations and CNVs) of the nine regulators of mt-rRNA modification were very rare (≤1.2% for all) (**Supplementary Figure S3A**) in gliomas using the “cBioPortal” of TCGA. All these results suggested that the aberrant expression of mt-rRNA regulators has the potential to be a prognostic biomarker, which is not generated by genetic mutations.

Correlation analyses aimed at exploring the immunological value of regulators of mt-rRNA modification are critical in determining the potential of immunotherapy. Our findings revealed that MRM2, NSUN4, TFB1M, and TRMT2B were correlated significantly with most immunomodulators in glioma, whereas MRM1 and RPUSD4 were correlated negatively (**Figure 2A**). Enrichment of immune signatures in the TME of glioma was also estimated using the ssGSEA algorithm, and the appearance of most of the regulators of mt-rRNA modification was correlated with levels of TIIC infiltration (**Figure 2C**). Furthermore, expression of regulators of mt-rRNA modification was associated with major moieties associated with immune checkpoints in glioma, especially PD-L1, PD-1, CD80, CD274, TIM3, and IDO1 (**Supplementary Figure S3B**). In summary, the above results suggested that the aberrant expression of mt-rRNA regulators in gliomas was TME-specific, which indicates the potential value of mt-rRNA regulators as targets for tumor immunotherapy.

### Identification of Two Subtypes With Distinct Functional Annotations, Clinicopathological Features, and Clinical Outcomes

**Figure 3:**
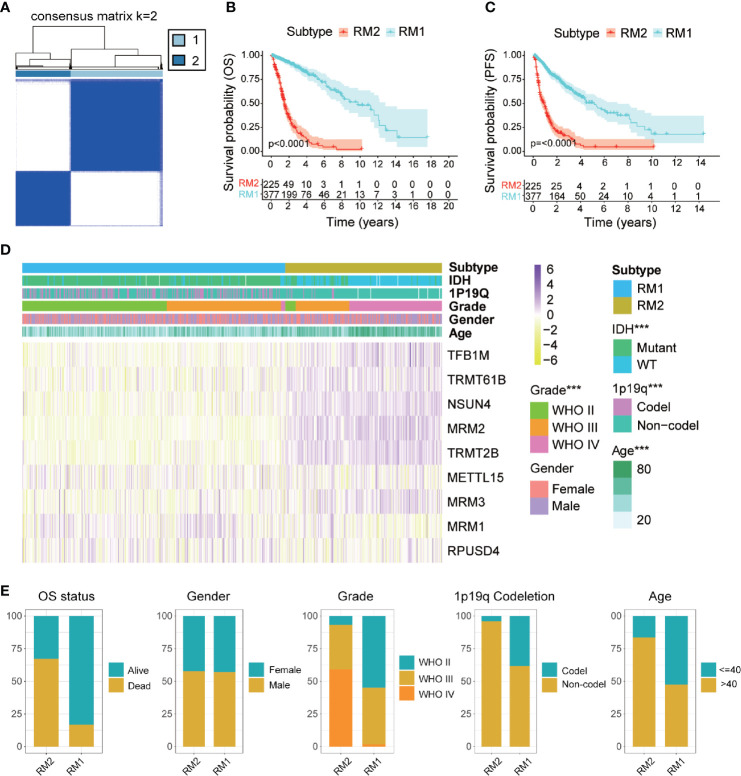
Comparisons of clinicopathologic features between two subtypes of mt-rRNA modification in TCGA cohort. **(A)** Consensus clustering matrix for the optimal number, k = 3. **(B, C)** Kaplan–Meier analyses for patients with the two subtypes of mt-rRNA modification. **(D)** Correlation between subtypes of mt-rRNA modification and clinicopathologic features. **(E)** Comparisons of OS status, gender, WHO grade, 1p19q codeletion, and age between two types of mt-rRNA modification. Significance: ***P < 0.001.

Glioma patients were classified into qualitatively different patterns of mt-rRNA modification based on the expression levels of nine regulators of mt-rRNA modification using the ConsensusClusterPlus algorithm. According to the relative change in the area under the CDF curve and the consensus heatmap, k = 2 seemed to be an adequate selection (**Figure 3A** and **Supplementary Figures S3C, D**). Hence, two distinct subtypes were identified: mt-rRNA modification 1 (RM1, n = 377, 62.6%) and mt-rRNA modification 2 (RM2, n = 225, 37.4%). Prognostic analysis for the two subtypes revealed that RM1 had a particularly significant survival advantage over RM2 in OS and PFS (**Figures 3B, C**). Subsequently, the clinicopathologic features of glioma patients with the two subtypes were compared (**Figure 3D** and **Supplementary Table S2**). The proportion of surviving patients (83%), younger in age at the diagnosis (53%), and 1p19q-codeletion status (38%) in RM1 were significantly higher than that for RM2 (surviving patients, 33%; younger in age at the diagnosis, 16%; 1p19q-codeletion status, 4%). Moreover, patients with the RM2 subtype had a higher WHO grade (P < 0.0001). However, there was no difference in gender between the two subtypes (**Figure 3E**).

**Figure 4:**
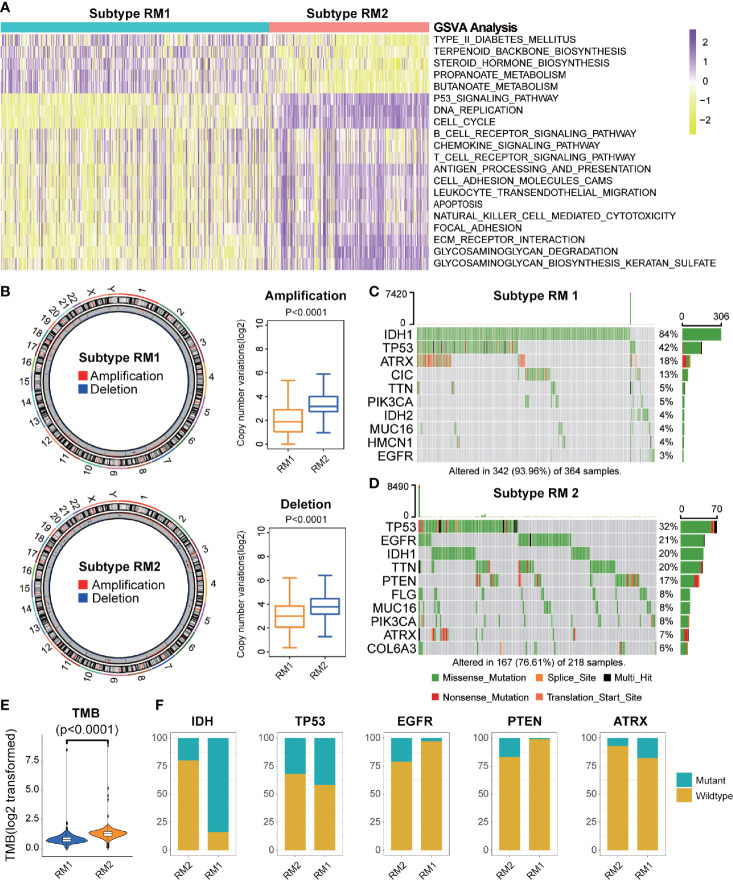
Functional annotation and genomic variations of two subtypes of mt-rRNA modification. **(A)** Heatmap of the top-20 differentially enriched molecular pathways between two types of mt-rRNA modification (yellow = high score and purple = low score). **(B)** Left: Circos plots illustrating the amplification and deletion of two subtypes. Right: Significant difference in CNV frequencies between two subtypes. **(C, D)** The waterfall plots illustrate the top-10 somatic mutations in tumors for two subtypes. **(E)** The different TMB between the two subtypes. **(F)** Comparisons of IDH, TP53, EGFR, PTEN, and ATRX mutations between two subtypes.

GSVA was conducted to explore the molecular pathways and potential mechanisms related to the two subtypes of mt-rRNA modification of glioma patients. We identified 135 differentially enriched molecular pathways: 36 pathways enriched in RM1 and 99 pathways enriched in RM2 (**Supplementary Table S3**). RM1-subtype tumors were correlated mainly with the tumor metabolism-related mechanisms (e.g., PROPANOATE_METABOLISM and BUTANOATE_METABOLISM) and pathway (e.g., “MTOR_SIGNALING_PATHWAY”). RM2-subtype tumors were correlated mainly with the genesis and development of tumors (e.g., “focal adhesion and apoptosis”), cancer-related signaling pathways (e.g., “P53 signaling pathway”, “cell cycle and chemokine signaling pathway”), and immune responses (e.g., “T and B cell receptor signaling pathway”, “antigen processing and presentation”, and “natural killer cell-mediated cytotoxicity”) (**Figure 4A**).

### Differences in Genomic Variation Between the Two Subtypes

Considering the indispensable role of genomic variation in the infiltration pattern of immune cells and regulation of tumor immunity, we undertook analyses of CNV and somatic mutation to explore the distinct genomic alterations in the different subtypes of mt-rRNA modification. The frequency of CNV, both amplification (P < 0.001) and deletion (P < 0.001), in patients with RM2, was significantly higher than that in patients with RM1 (**Figure 4B**). Afterwards, a “waterfall” map of somatic mutations showed that each subtype of mt-rRNA modification had specific mutated genes (**Figures 4C, D**). The proportion of RM2-subtype patients with mutations of IDH1 (20%), ATRX (7%), EGFR (21%), and PTEN (17%) was significantly different from those with the RM1 subtype (P < 0.01 for all), whereas there was no significant difference in the mutation frequency of TP53 (**Figure 4F**). Moreover, patients tended to bear a higher TMB in the RM2 subtype than that in the RM1 subtype (P < 0.0001) (**Figure 4E**). Taken together, these findings suggested that glioma patients with a different subtype of mt-rRNA modification could have differences in response to immunotherapy.

### Distinct Immunological Characteristics and Immunotherapy of the Two mt-rRNA Modification Subtypes

**Figure 5:**
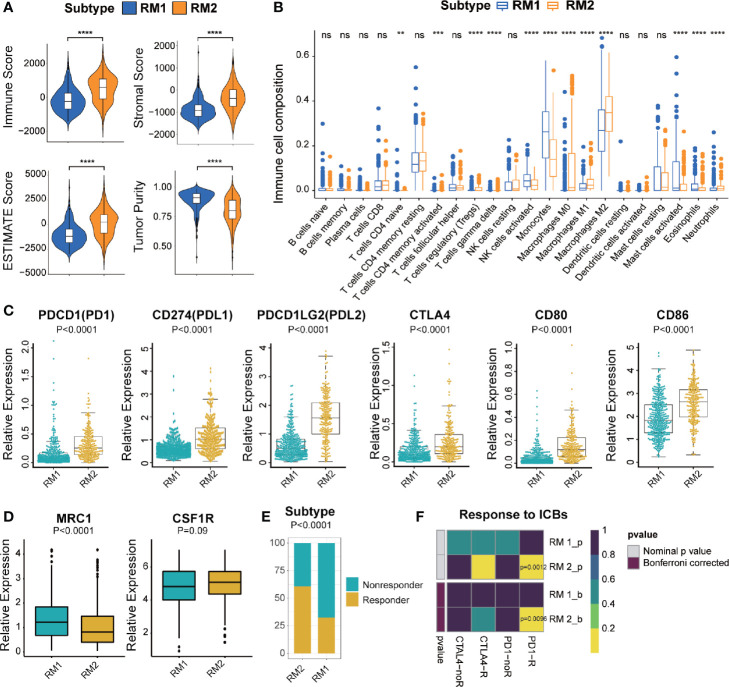
Different TME characteristics and response to immunotherapy of two subtypes in TCGA cohort. **(A)** Comparisons of immune score, stromal score, tumor purity, and ESTIMATE score between two subtypes. **(B)** Difference in the abundance of TIICs between two subtypes. **(C, D)** Different levels of ICBs between two subtypes. **(E)** Different proportions of responders and non-responders to immunotherapy between two subtypes. **(F)** Prediction of response to ICBs (PD1 and CTLA4) therapy in different subtypes. Significance: ns *P* > 0.05, < 0.05, **P < 0.01, ***P < 0.001, ****P < 0.0001.

To investigate the immunologic characteristics of glioma patients, we first used the ESTIMATE algorithm to reveal the TME compositions of the two subtypes of mt-rRNA modification. Compared with patients with the RM1 subtype, patients with the RM2 subtype possessed higher stromal, immune, and ESTIMATE scores, accompanied by lower tumor purity (P < 0.0001 for all) (**Figure 5A**). Afterwards, the CIBESORT algorithm was used to quantify the TIIC abundance of glioma patients. Most subsets of CD4+T cells and macrophages as well as neutrophils infiltrated in the RM2 subtype, whereas activated natural killer (NK) cells, mast cells, monocytes, and eosinophils infiltrated in the RM1 subtype (**Figure 5B**). These analyses suggested that the RM2 subtype was associated with higher levels of TIIC infiltration and lower tumor purity, which could have implications for immunotherapy.

Most immune-checkpoint molecules were significantly different between the two subtypes, among which expression of PD1, CTLA, and their ligands (PD-L1, PD-L2, CD80 and CD86, P < 0.0001 for all) was significantly higher in the RM2 subtype (**Figure 5C**). Moreover, we also investigated the correlation between the marker of M2-type (MRC1/CD206 and CSF1R/CD115) and different mt-rRNA subtypes. It revealed that the expression of MRC1 was significantly lower in the RM2 subtype, while CSF1R represented no difference (**Figure 5D**). The results indicated that macrophages were mainly polarization toward the M1-type state (which markers are CD80 and CD86) in the RM2 subtype. Based on these results, we used the TIDE algorithm to predict the potential response to immunotherapy in glioma patients. The number of immunotherapy responders with the RM2 subtype was nearly twice that of immunotherapy responders with the RM1 subtype (61% vs. 32%, P < 0.01) (**Figure 5E**). Then, subclass mapping was utilized to predict the response of the two subtypes of mt-rRNA modification to ICB therapy (anti-PD1 and anti-CTLA4). Patients with the RM2 subtype were more sensitive to anti-PD1 treatment (Bonferroni P = 0.0096) (**Figure 5F**).

### Identification and Validation of a mt-rRNA Regulator Predictor

**Figure 6:**
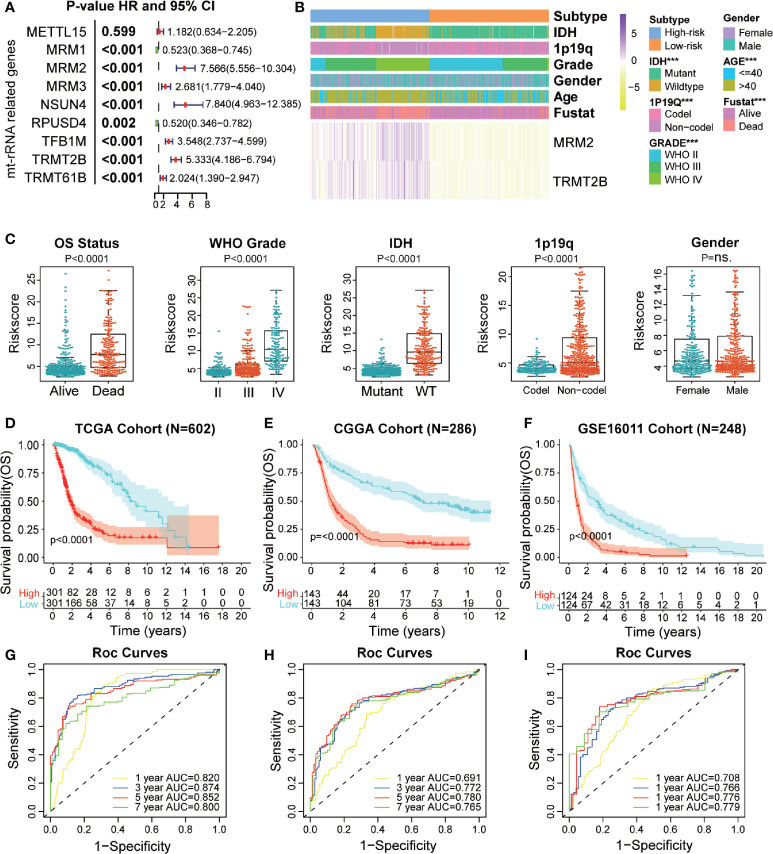
Prognostic value of a mt-rRNA predictor in gliomas. **(A)** Prognostic value of nine regulators of mt-rRNA modification in TCGA cohort. **(B)** Correlation between the mt-rRNA predictor and clinicopathologic features. **(C)** Comparisons of OS, WHO grade, 1p19q codeletion, IDH mutation, and gender between two risk subtypes of mt-rRNA. **(D–F)** Kaplan–Meier analyses of the mt-rRNA predictor with OS in TCGA, CGGA, and GSE16011 cohorts. **(G–I)** AUC of ROC curves for predicting 1-, 3-, 5-, and 7-year OS in TCGA, CGGA, and GSE16011 cohorts, respectively. Significance: ns *P* > 0.05, ***P < 0.001.

We used bulk RNA-sequencing data from four independent glioma cohorts (TCGA, CGGA, GSE16011, and SAHNU databases) to explore the value of these mt-rRNA regulators for clinical application. First, in the training set, we found eight mt-rRNA regulators to be correlated significantly with OS (P < 0.01), which was consistent with our predictions using the GEPIA2 website (**Figure 6A**). Among these genes, MRM2, MRM3, NUSN4, TFB1M, TRMT2B, and TRMT61B acted as protective factors, whereas MRM1 and RPUSD4 acted as risk factors. Hence, the LASSO Cox regression algorithm was employed to the relevant patterns of mt-rRNA modification *via* expression of the most critical eight regulators in the TCGA cohort, which acted as the training set (**Supplementary Figures S3E, F**). Two regulators of mt-rRNA modification (MRM2 and TRMT2B) were selected to construct the model of risk prediction. The formula used for calculation of the mt-rRNA score was: mt-rRNA score = 0.108 × (MRM2 expression) + 0.125 × (TRMT2B expression). According to the median mt-rRNA score, glioma patients were divided into a low-risk subtype and a high-risk subtype in TCGA, CGGA, GSE16011, and SAHNU cohorts. Afterwards, we explored the association and demographic features between the mt-rRNA score and each clinicopathologic feature (**Figure 6B** and **Supplementary Table S4**). The mt-rRNA score was significantly different between glioma patients stratified by OS status, WHO grade, 1p19q codeletion, and IDH status, but not by gender in the training set (**Figure 6C**). Similar results were observed in the validation sets, CGGA cohort, and GSE16011 cohort (**Supplementary Figures 4A, B**). In addition, we explored relative mRNA expression of MRM2 and TRMT2B using RT-qPCR. Results revealed that MRM2 and TRMT2B were differentially expressed between NBTs, low-grade glioma tissues, and high-grade glioma tissues (**Supplementary Figure 5B**). We also found the mt-rRNA score to be correlated significantly with WHO grade (P = 0.004) and age (P = 0.008) in the SAHNU cohort (**Supplementary Figure 5A, C**).

Moreover, glioma patients with the low-risk subtype presented significantly better OS (P < 0.0001) (**Figure 6D**) and PFS (P < 0.0001; **Supplementary Figure 5D**) in the training set. ROC analyses revealed the predictive efficiency at 1, 3, 5, and 7 years (area under the ROC curve (AUC >0.75 for all) for distinguishing the low-risk subtype and high-risk subtype in the training set (**Figure 6G** and **Supplementary Figure S5E**). Consistently, the mt-rRNA predictor had excellent performance in discriminating the outcomes (**Figures 6E, F**) for glioma patients as evaluated in the two validation sets, with powerful predictive efficiency (AUC >0.7 for both) (**Figures 6H, I**). We also explored the prognostic value of the mt-rRNA predictor for different WHO grades. Survival analyses using the Kaplan–Meier method demonstrated that patients with lower-grade glioma and glioblastoma (GBM) patients with low-risk scores presented significantly better OS in the CGGA and GSE16011 cohorts (**Supplementary Figures S6C, D**), data which were consistent with the results in TCGA cohort (**Supplementary Figure S6A**). However, there was no difference in PFS between glioblastoma patients stratified by the mt-rRNA score in the TCGA cohort (**Supplementary Figure S6B**). Univariate and multivariate Cox regression analyses for the three cohorts revealed significant associations between the mt-rRNA predictor and OS/PFS in TCGA, CGGA, and GSE16011 cohorts (P < 0.05 for all) (**Supplementary Figure S7A–D**), which indicated that the mt-rRNA predictor was an independent prognostic indicator. Taken together, these results indicated that the mt-rRNA predictor calculated using the mt-rRNA score could predict the prognosis and clinicopathologic features of glioma patients accurately.

### The High-Risk Subtype Exhibited Greater Sensitivity to Immunotherapy but Was Resistant to Temozolomide

**Figure 7:**
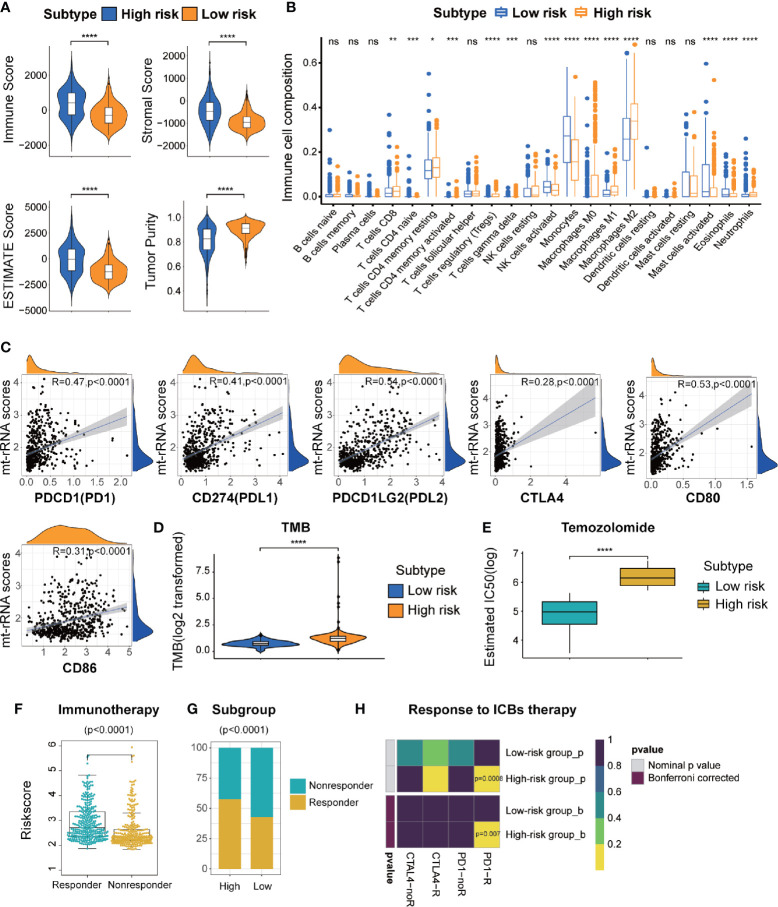
Different TME characteristics, immunotherapy, and response to temozolomide therapy of two risk subtypes of mt-rRNA in TCGA cohort. **(A)** Comparisons of immune score, stromal score, tumor purity, and ESTIMATE score between high-risk and low-risk subtypes. **(B)** Different abundance of TIICs between high-risk and low-risk subtypes. **(C)** Correlation analyses between the mt-rRNA score and ICBs. **(D)** Comparison of the TMB between high-risk and low-risk subtypes. **(E)** Different IC50 of temozolomide between two subtypes in the GDSC database. **(F)** Comparison of the mt-rRNA score between the responder group and non-responder group. **(G)** Different proportions of responders and non-responders to immunotherapy between two subtypes. **(H)** Prediction of response to ICBs (PD1 and CTLA4) therapy in high-risk and low-risk subtypes. Significance: ns *P* > 0.05, *P < 0.05, **P < 0.01, ***P < 0.001, ****P < 0.0001.

First, TME patterns were also estimated in the CGGA and GSE16011 cohorts using the same method. Similar to the results of TCGA (**Figure 7A**), the stromal, immune, and ESTIMATE scores were significantly higher and the tumor purity was lower in the high-risk subtype (P < 0.05 for all), which indicated the high abundance of stromal cells and immune cells and low tumor purity in the high-risk subtype (**Supplementary Figures S8A, C**). CIBERSORT analyses further revealed that memory resting T cell CD4, resting NK cells, and most macrophage subsets were infiltrated significantly in the high-risk subtype, whereas activated NK cells and mast cells were abundant in the low-risk subtype. (**Figure 7B** and **Supplementary Figures S8B, D**). Subsequently, we also investigated the relationship between the expression of immune-checkpoint molecules and mt-rRNA score in the three cohorts. We found most of the immune-checkpoint molecules were associated with the mt-rRNA score (**Supplementary Figures S7A–C**). For the well-known ICBs, the expression of PD1 and its ligands PD-L1 and PD-L2 was correlated significantly with the mt-rRNA score, but the correlation between CTLA4 and the mt-rRNA score was poor (**Figure 7C**). These ICBs were also expressed differently between the two subtypes in the validation sets (**Supplementary Figures S9D, E**), with the exception of the ligands of CTLA4 in the GSE16011 cohort (**Supplementary Figure S9E**). Moreover, patients tended to bear a higher TMB in the high-risk subtype than that in the low-risk subtype (P < 0.0001, **Figure 7D**).

On the basis of the above findings, the TIDE algorithm was employed to predict the response to ICB therapy of glioma patients in the three cohorts. The mt-rRNA score of responders and non-responders was significantly different (P < 0.0001 for all) (**Figure 7F** and **Supplementary Figures S10A, D**). Moreover, the proportion of responders to ICB therapy who had the high-risk subtype was significantly higher than the proportion of responders to ICB therapy who had the low-risk subtype (P < 0.0001 for all) (**Figure 7G** and **Supplementary Figures S10B, E**). Subclass mapping demonstrated that glioma patients with the high-risk subtype were more sensitive to anti-PD1 therapy in the TCGA, CGGA, GSE16011 cohort, with Bonferroni P = 0.007, 0.032, and 0.046, respectively (**Figure 7H** and **Supplementary Figures S10C, F**).

Considering that temozolomide chemotherapy is the first-line treatment for glioma, we explored the response of patients with the two subtypes of mt-rRNA risk to temozolomide therapy based on the GDSC database. The log-IC50 of temozolomide in patients with the low-risk subtype was significantly lower than that of cases with the high-risk subtype. Hence, glioma patients with the high-risk subtype were more resistant to temozolomide than those with the low-risk subtype (**Figure 7E**).

## Discussion

In this study, we conducted comprehensive analyses of the correlation between the patterns of mt-rRNA modification and efficacy of immunotherapy and chemotherapy in glioma, proposed a predictor to distinguish subtypes based on the mt-rRNA regulator, and verified this predictor with available RNA-sequencing data and related clinical information from four independent cohorts. First, we integrated the expression of 122 immunomodulators, 29 immune signatures calculated with the ssGSEA, clinical outcomes, clinicopathologic characteristics, genomic variations, and the nine mt-rRNA regulators of glioma patients in the TCGA database, and studied the relationship between them. Then, the TME and composition of TIICs were evaluated by ESTIMATE and CIBERSORT algorithms, respectively. We divided glioma patients into two subtypes of mt-rRNA-modification patterns based on the expression profiles of nine mt-rRNA regulators and clarified the association between the two subtypes and clinicopathologic parameters. Glioma patients with RM2 could achieve better therapeutic effects through immunotherapy (especially anti-PD-1 therapy) as predicted by the TIDE algorithm. In addition, patients in this group had longer OS and PFS. To make the method more clinically practical for glioma patients, the two most critical prognostic genes for mt-rRNA regulation were identified and defined as mt-rRNA predictors to distinguish different mt-rRNA subtypes by using the LASSO algorithm. The forecasted effect of this predictor was verified further in the CGGA, GSE16011, and SAHNC cohorts.

In recent years, immunotherapy for tumors has been studied extensively using ICBs, tumor-cell immunotherapy, and antitumor vaccines. However, the immunotherapy effect in phase-III clinical trials for glioblastoma has not been satisfactory (24, 43). Several factors can determine the efficacy of immunotherapy in glioma patients. Taking anti-PD-1/PD-L1 treatment as an example, the TMB, mismatch repair, expression of PD-L1 (CD274), and TICC composition can affect efficacy (25, 44, 45). When investigating immunotherapy in phase-III clinical trials for glioblastoma, patients are not screened for these parameters, and such undifferentiated treatment may be the reason for the poor efficacy of immunotherapy. Hence, comprehensive analyses of the role of these factors will help to enhance understanding of the immune response to gliomas and guide more efficacious immunotherapy strategies.

Increasing evidence has demonstrated that RNA modifications have indispensable roles in inflammation, innate immunity, and anti-tumor activities, among which the most extensive and in-depth research has been on m6A. Bo Zhang and colleagues demonstrated that modification of m6A methylation is involved in the regulation of the microenvironment of gastric cancer, and has a guiding role in immunotherapy (46). Jianyang Du and coworkers revealed that m6A regulators had different modification patterns and characteristics of immunity in low-grade glioma (47). Most studies have focused on m6A modification, the overall TME characterization mediated by mt-rRNA modification and its regulators is not recognized.

In this work, based on nine mt-rRNA regulators, we revealed two distinct subtypes of mt-rRNA modification with significantly different TME characterization. RM2 was characterized by the activation of immunity and related enriched pathways, which corresponded to an immune-activated phenotype. RM1 was characterized by infiltration of innate immune cells (eosinophils, NK cells, and mast cells), which corresponded to an immune-excluded phenotype (48, 49). Meanwhile, patients with this pattern of mt-rRNA modification showed a matching survival advantage. Recent studies have suggested that tumors with immune-excluded phenotype also show infiltration of immune cells because the latter remain in the matrix around the tumor cell “nests” rather than penetrating the parenchyma of tumor cells (50, 51). Moreover, we revealed a significant correlation between the subtypes and TMB, including several common somatic mutations (TP53, PTEN, and EGFR) in gliomas (52). mt-rRNA modification played an important part in formation of the “immune landscape” of the TME, which suggests that mt-rRNA modification may affect the therapeutic efficacy of ICBs. Glioma patients with the RM2 subtype had a significantly better response to anti-PD-1 immunotherapy. We created an mt-rRNA predictor that could be used to characterize cell infiltration in the TME, TMB, and clinicopathologic features of glioma patients. This mt-rRNA predictor could act as an independent prognostic biomarker for predicting clinical outcomes as well as the efficacy of PD-1 immunotherapy and temozolomide chemotherapy through the mt-rRNA score.

However, our study still had two main limitations. First, we obtained only 33 glioma specimens with pathologic data, but no clinical outcomes, to verify the results of our study. We need to collect more glioma samples and obtain the corresponding clinical outcomes from our center in the future. Second, our conclusions are based only on the analysis and prediction of transcriptome data in publicly available databases, the relationship between mt-rRNA modification and immunotherapy responsiveness needs to be tested in the future immunotherapy cohort.

## Conclusions

In conclusion, according to patterns of mt-rRNA modification, glioma patients can be divided into two distinct subtypes which have a notably different prognosis and response to immunotherapy. Our study provides a potential strategy for “individualized” tumor immunotherapy in the future. We constructed a mt-rRNA predictor which made subgrouping based on mt-rRNA regulators and precision immunotherapy clinically feasible.

## Data Availability Statement

The datasets presented in this study can be found in online repositories. The names of the repository/repositories and accession number(s) can be found in the article/**Supplementary Material**.

## Ethics Statement

The studies involving human participants were reviewed and approved by The medical ethics committee of the Second Affiliated Hospital of Nanchang University. The patients/participants provided their written informed consent to participate in this study.

## Author Contributions

XZ and KH designed the research. JL, MW, and MY contributed to the collect the brain tissue samples and matching clinical data. PW contributed to the public data collection and analysis, figures and tables, complete the experiments, and were involved in manuscript writing. XZ and KH performed the correction of the language and revision. All authors contributed to the article and approved the submitted version.

## Funding

The present study was supported by the National Natural Science Foundation of China (grant nos. 81960456, 82002660, 81760445, and 81760446).

## Conflict of Interest

The authors declare that the research was conducted in the absence of any commercial or financial relationships that could be construed as a potential conflict of interest.

## Publisher’s Note

All claims expressed in this article are solely those of the authors and do not necessarily represent those of their affiliated organizations, or those of the publisher, the editors and the reviewers. Any product that may be evaluated in this article, or claim that may be made by its manufacturer, is not guaranteed or endorsed by the publisher.
