# Supplementary figures and images for: Human Mitochondrial Ribosomal RNA Modification-Based Classification Contributes to Discriminate the Prognosis and Immunotherapy Response of Glioma Patients
Source: Front Immunol. 2021 Sep 9;12:722479. doi: 10.3389/fimmu.2021.722479 (PMC8458820; doi:10.3389/fimmu.2021.722479)

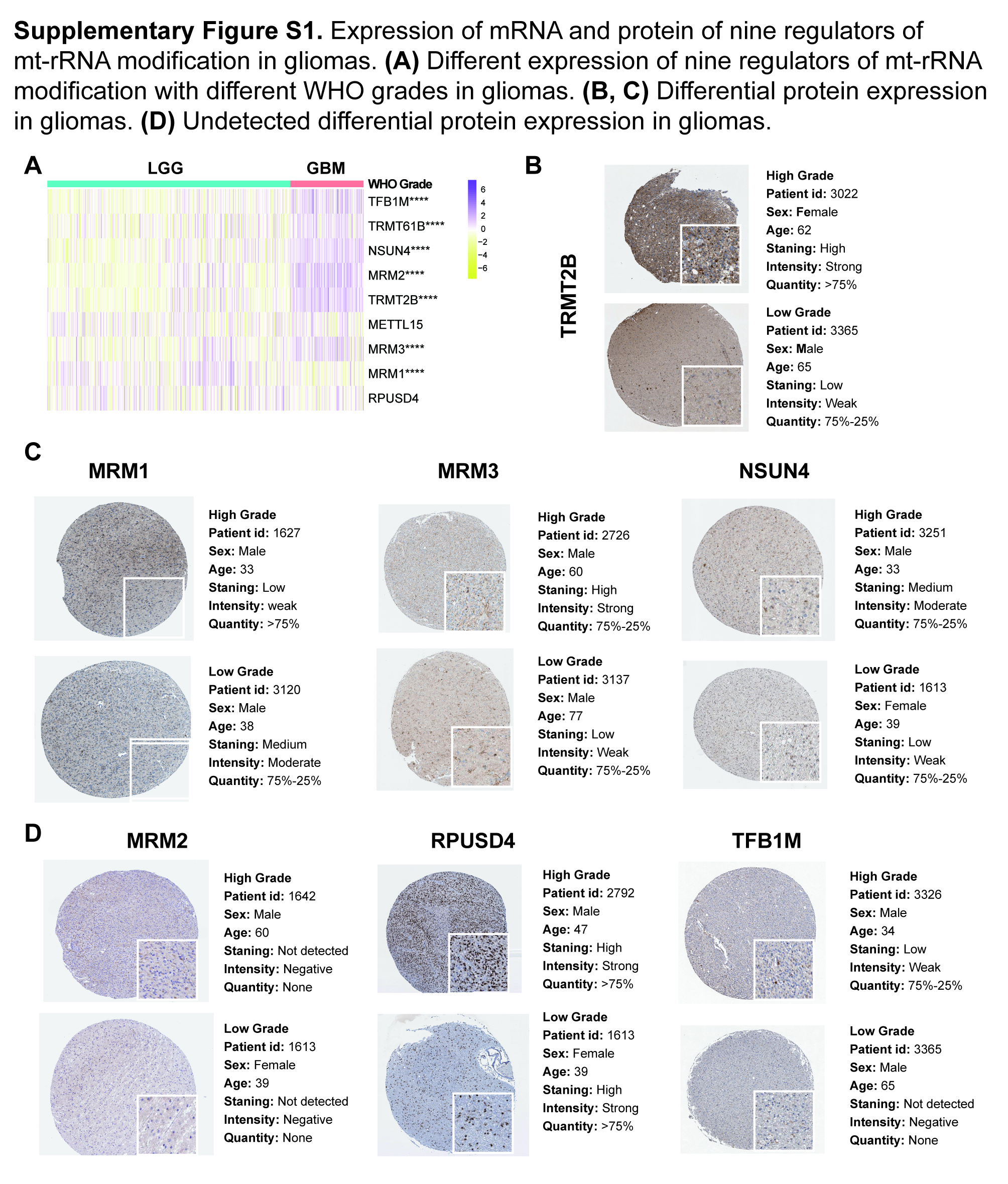

Supplement: Supplementary file 1 [file Image_1.tif]

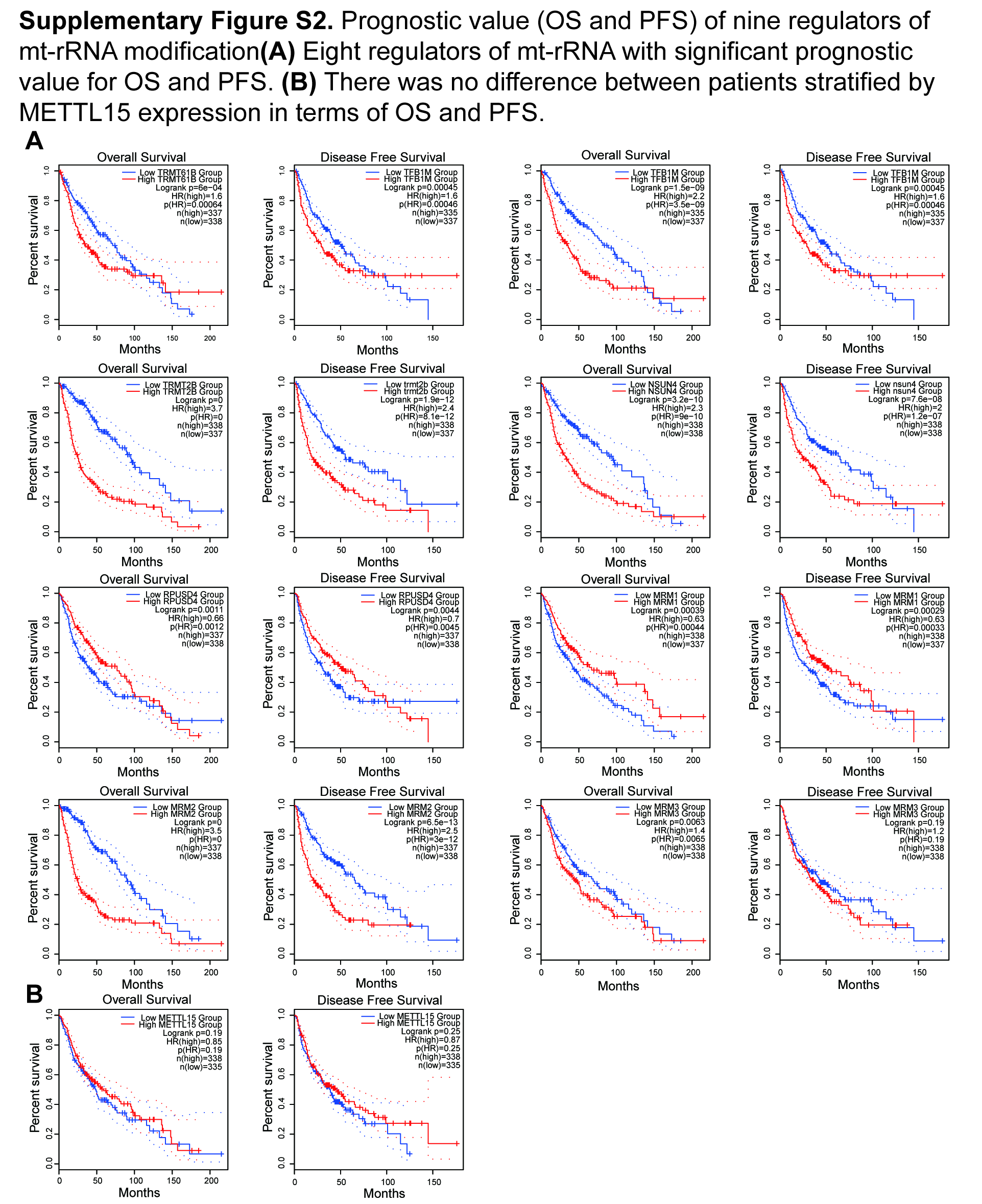

Supplement: Supplementary file 2 [file Image_2.tif]

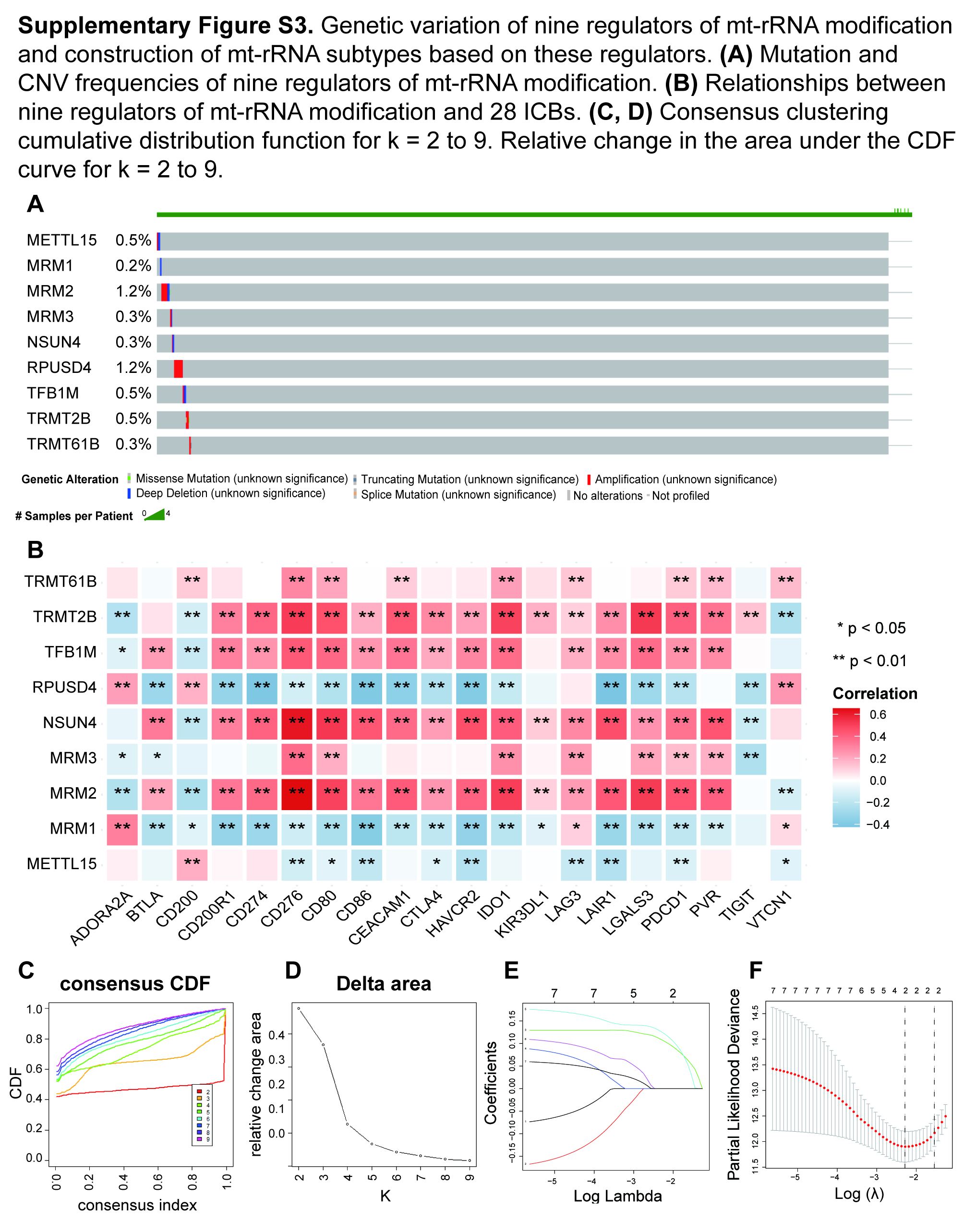

Supplement: Supplementary file 3 [file Image_3.tif]

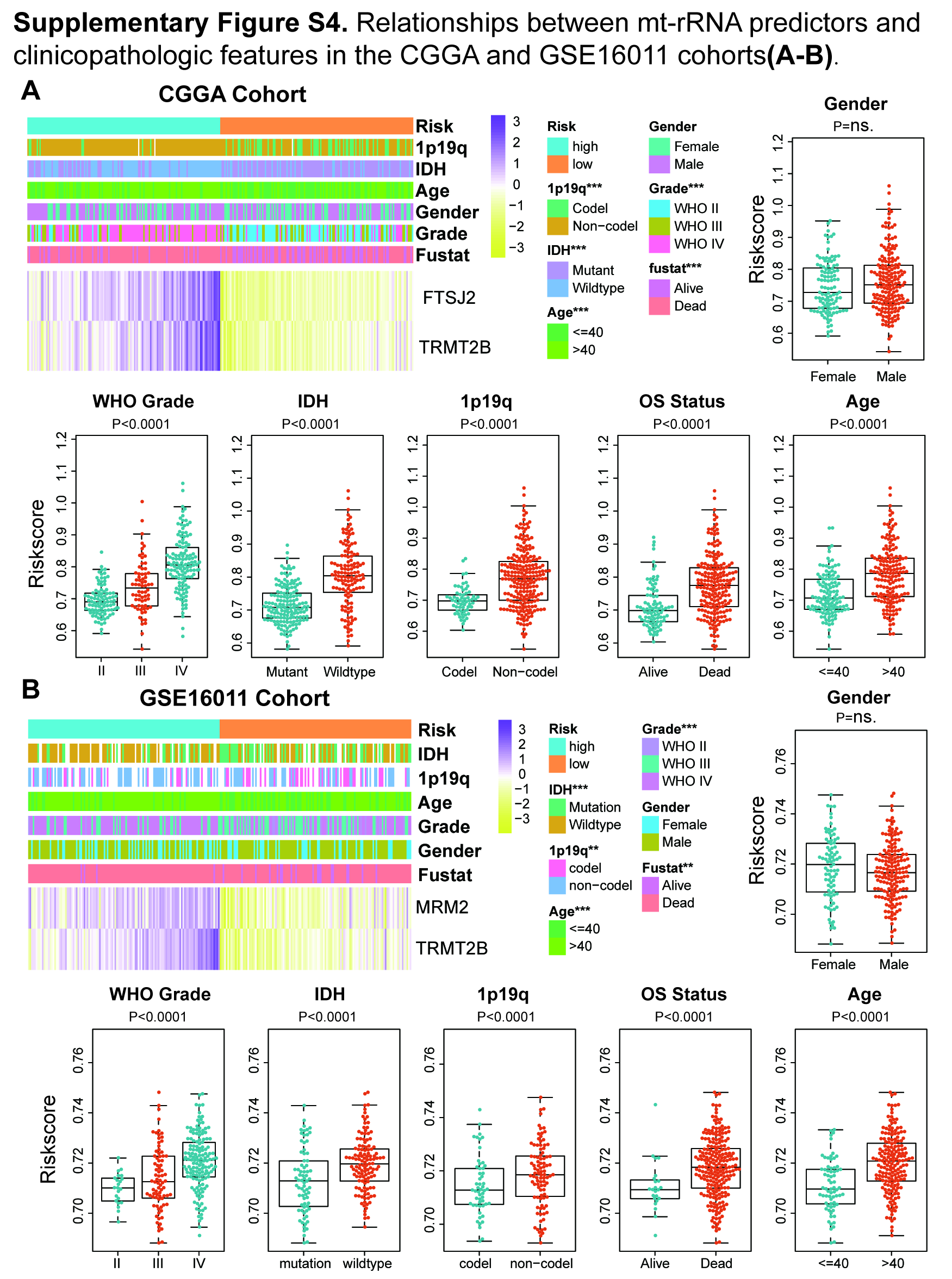

Supplement: Supplementary file 4 [file Image_4.tif]

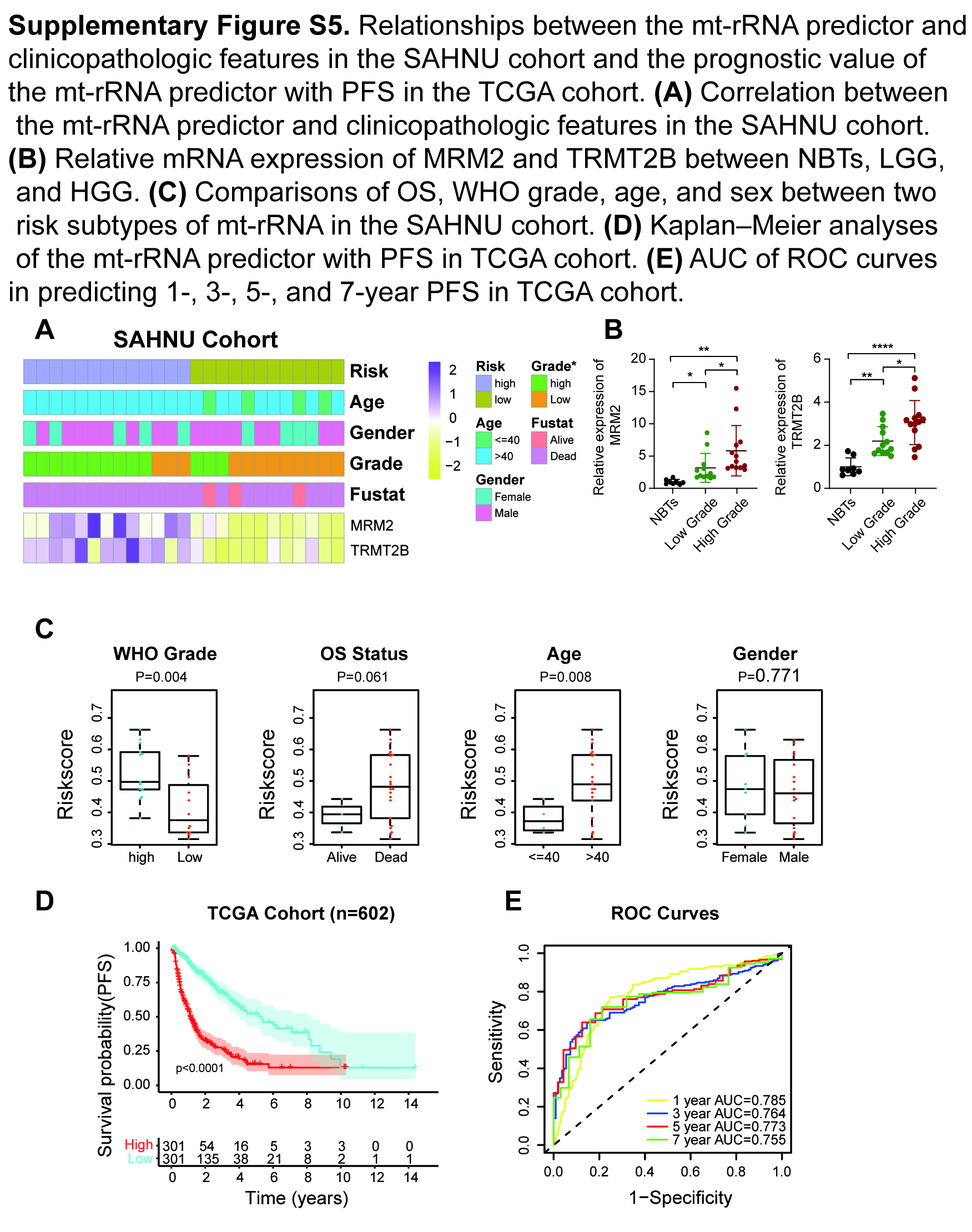

Supplement: Supplementary file 5 [file Image_5.tif]

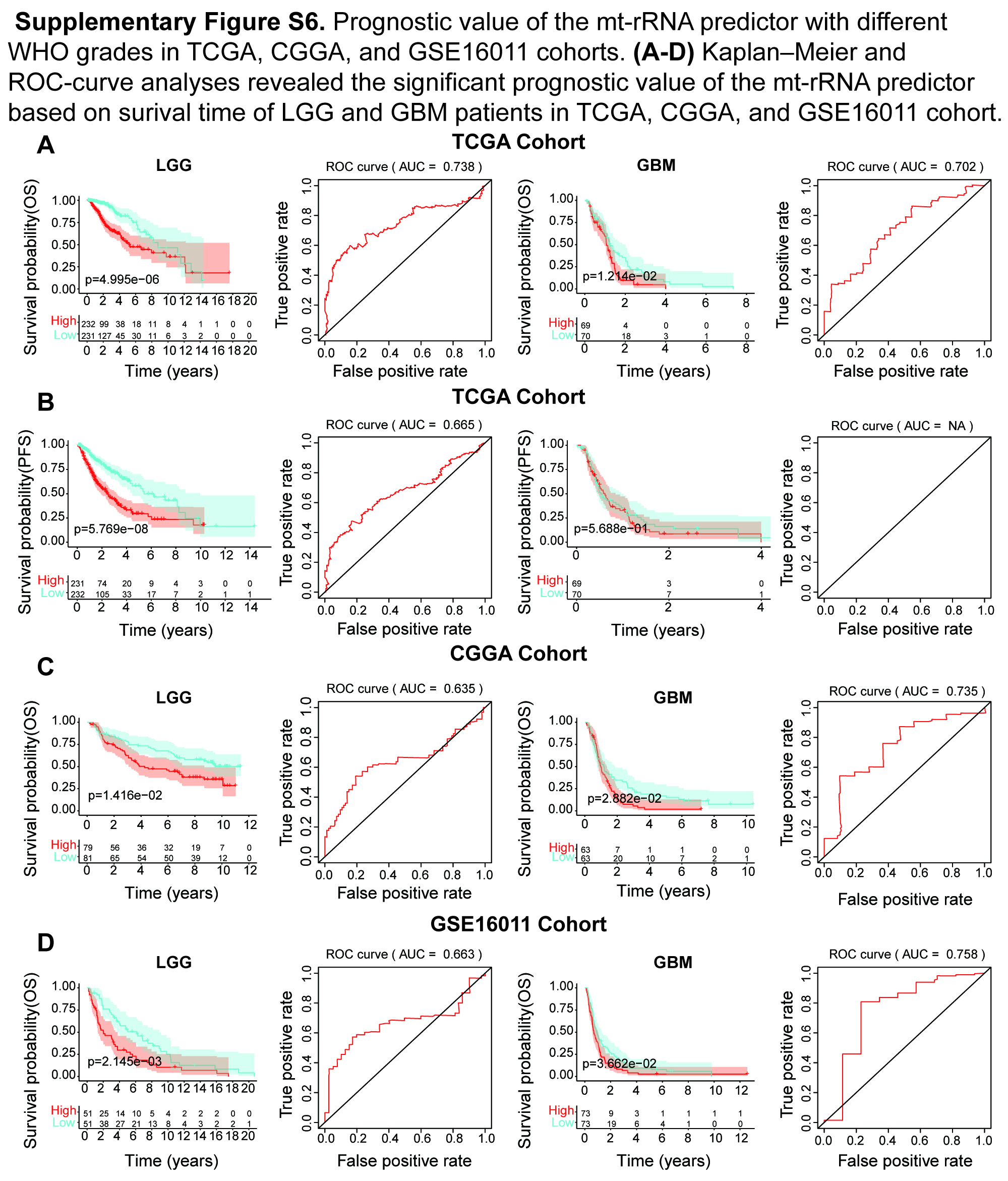

Supplement: Supplementary file 6 [file Image_6.tif]

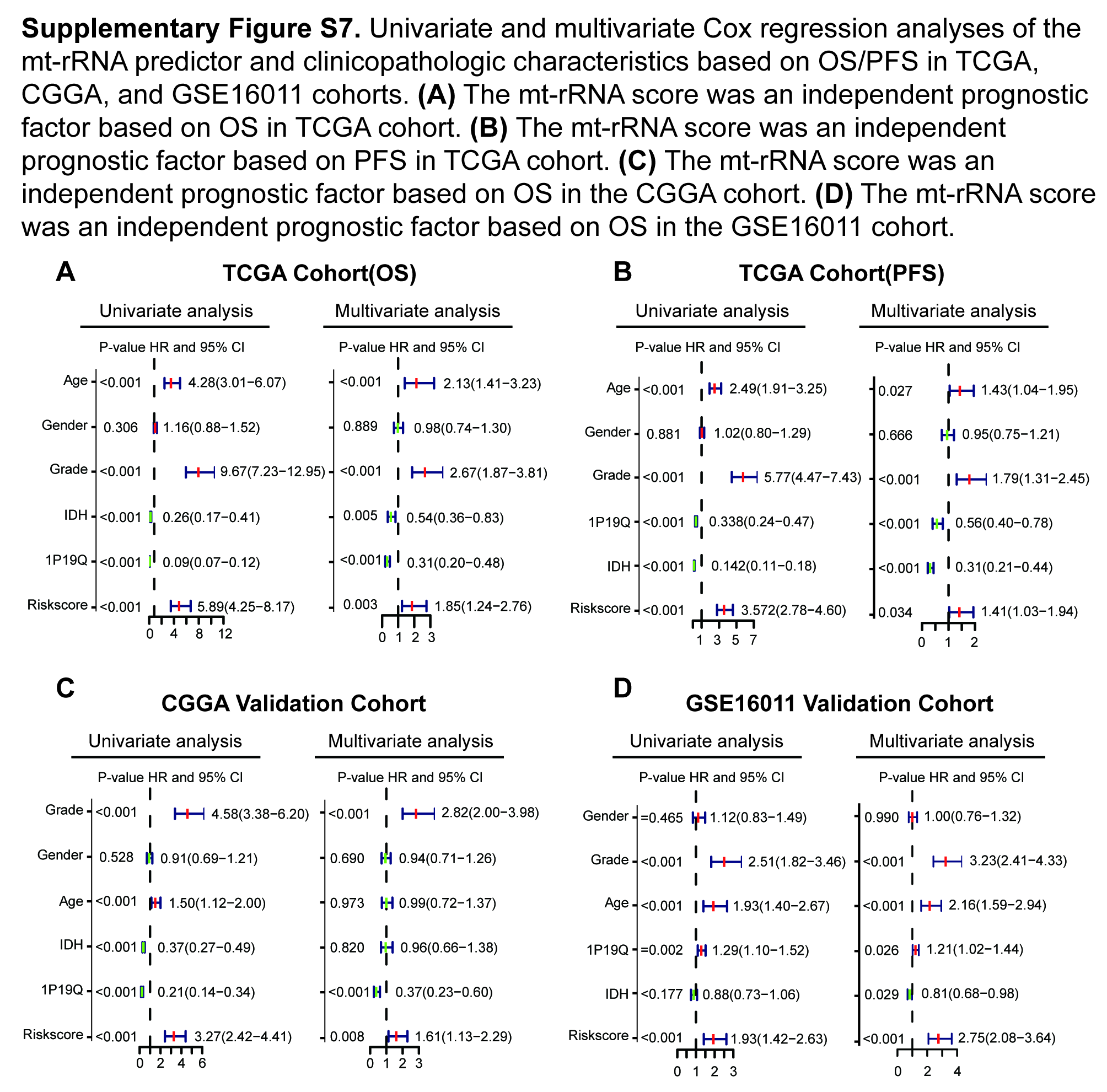

Supplement: Supplementary file 7 [file Image_7.tif]

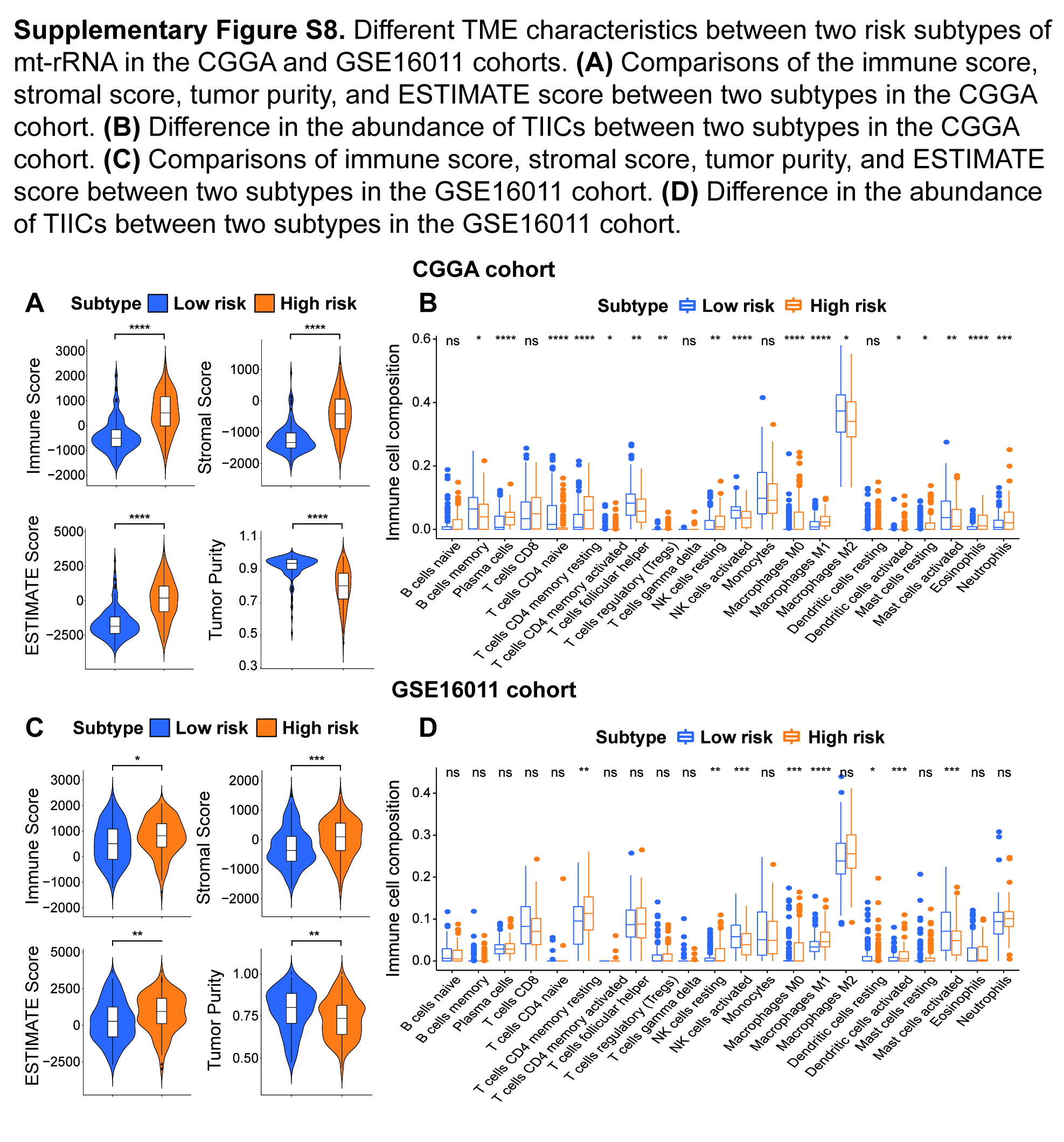

Supplement: Supplementary file 8 [file Image_8.tif]

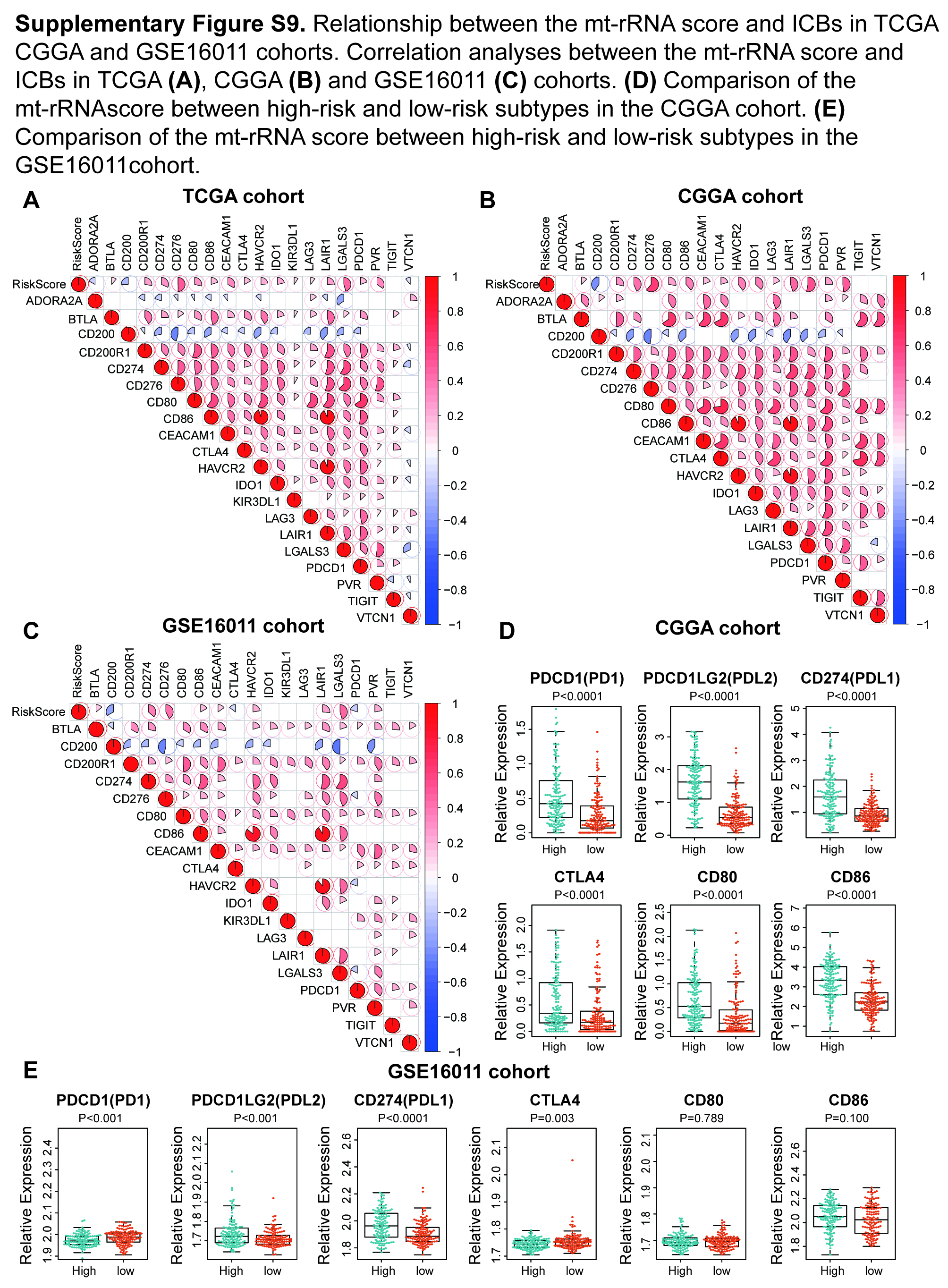

Supplement: Supplementary file 9 [file Image_9.tif]

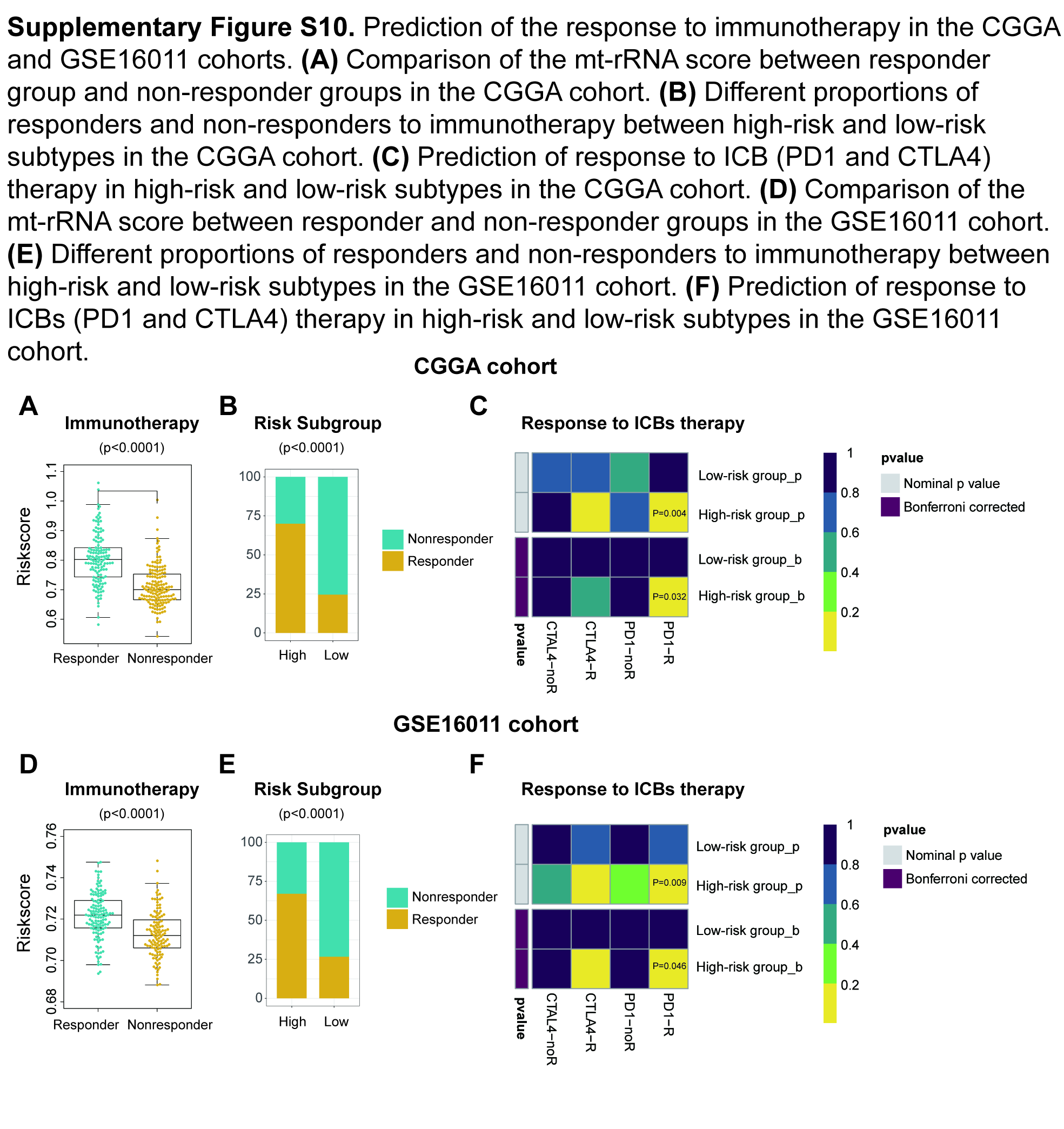

Supplement: Supplementary file 10 [file Image_10.tif]
